# Leflunomide-Induced Severe Cutaneous Adverse Reaction, Severe Drug-Induced Liver Injury With Hepatic Encephalopathy, and Hemophagocytic Lymphohistiocytosis: A Case Report

**DOI:** 10.7759/cureus.115383

**Published:** 2026-08-28

**Authors:** Kiran Prasad Moparthi, Satyavani Jampani

**Affiliations:** 1 Internal Medicine, Focus Hospital, Hyderabad, IND; 2 Dermatology, Focus Hospital, Hyderabad, IND

**Keywords:** acute liver failure, dress syndrome, hemophagocytic lymphohistiocytosis, leflunomide, rheumatoid arthritis

## Abstract

Leflunomide, a disease-modifying antirheumatic drug (DMARD) employed for the treatment of rheumatoid arthritis, generally holds a reputation for safety. However, it occasionally induces severe adverse reactions, including acute liver failure, drug reaction with eosinophilia and systemic symptoms (DRESS) syndrome, and hemophagocytic lymphohistiocytosis (HLH). This report details the case of a 46-year-old female who developed these complications shortly after commencing leflunomide therapy, manifesting severe cutaneous adverse reactions, severe drug-induced liver injury with Grade I hepatic encephalopathy, and subsequently HLH. Her clinical journey underscores the critical need for vigilance and prompt management in patients showing signs of severe drug-induced reactions. Following the HLH-94 treatment protocol, her condition improved significantly, highlighting the efficacy of immediate and appropriate intervention. This case not only exemplifies the potential severe complications associated with leflunomide but also illustrates the life-saving potential of early diagnosis and treatment in cases of drug-induced HLH and DRESS syndrome. This case contributes to the literature by underscoring the complexities of managing rare but severe side effects of commonly used medications.

## Introduction

Leflunomide is an immunomodulatory disease-modifying antirheumatic drug (DMARD) used to manage rheumatoid arthritis​​ [1]. Despite its general safety profile, leflunomide has been associated with rare but severe systemic toxicities. Managing these adverse reactions is uniquely complicated by the active metabolite's prolonged elimination half-life of up to two weeks, which necessitates emergency accelerated clearance techniques such as a cholestyramine washout procedure. Among these severe reactions, the drug has been linked with severe acute drug-induced liver injury, drug reaction with eosinophilia and systemic symptoms (DRESS) syndrome, and secondary hemophagocytic lymphohistiocytosis (HLH)​ [2].

DRESS syndrome is a life-threatening, delayed hypersensitivity type IV reaction, characterized by a widespread rash, fever, eosinophilia, and internal organ involvement, often affecting the liver [3]​. The pathogenesis of DRESS involves a complex interplay among genetic predisposition, drug metabolism, and immune response, leading to a cytokine storm and hyperinflammatory state​​ [4]. This hyperinflammatory state can further complicate into HLH, a disorder of uncontrolled immune activation resulting in overwhelming systemic inflammation and multi-organ failure [5]​.

HLH itself is a severe, life-threatening condition that can be triggered by infections, malignancies, autoimmune diseases, and certain medications like leflunomide​​ [6]. It is characterized by fever, cytopenias, hepatosplenomegaly, hyperferritinemia, and hemophagocytosis on bone marrow biopsy​. Early diagnosis and prompt initiation of immunosuppressive therapy, following protocols like HLH-94, are crucial for improving outcomes in patients with HLH​.

This case report presents a rare instance of leflunomide-induced liver failure, DRESS syndrome, and subsequent HLH, emphasizing the importance of prompt recognition and management of these severe adverse reactions to prevent fatal outcomes. Cases of leflunomide-induced liver injury reported from India have been noted to differ clinically from Western cases, typically presenting with severe cutaneous reactions such as DRESS or Stevens-Johnson syndrome/toxic epidermal necrolysis, predominantly in women, with high associated morbidity and mortality--a pattern whose basis remains unexplained. This case directly bridges an important knowledge gap by adding to this unique, poorly characterized regional phenotype and further extends the literature by documenting progression to secondary HLH, an exceptionally rare and life-threatening complication within this clinical spectrum.

## Case presentation

**Figure 1:**
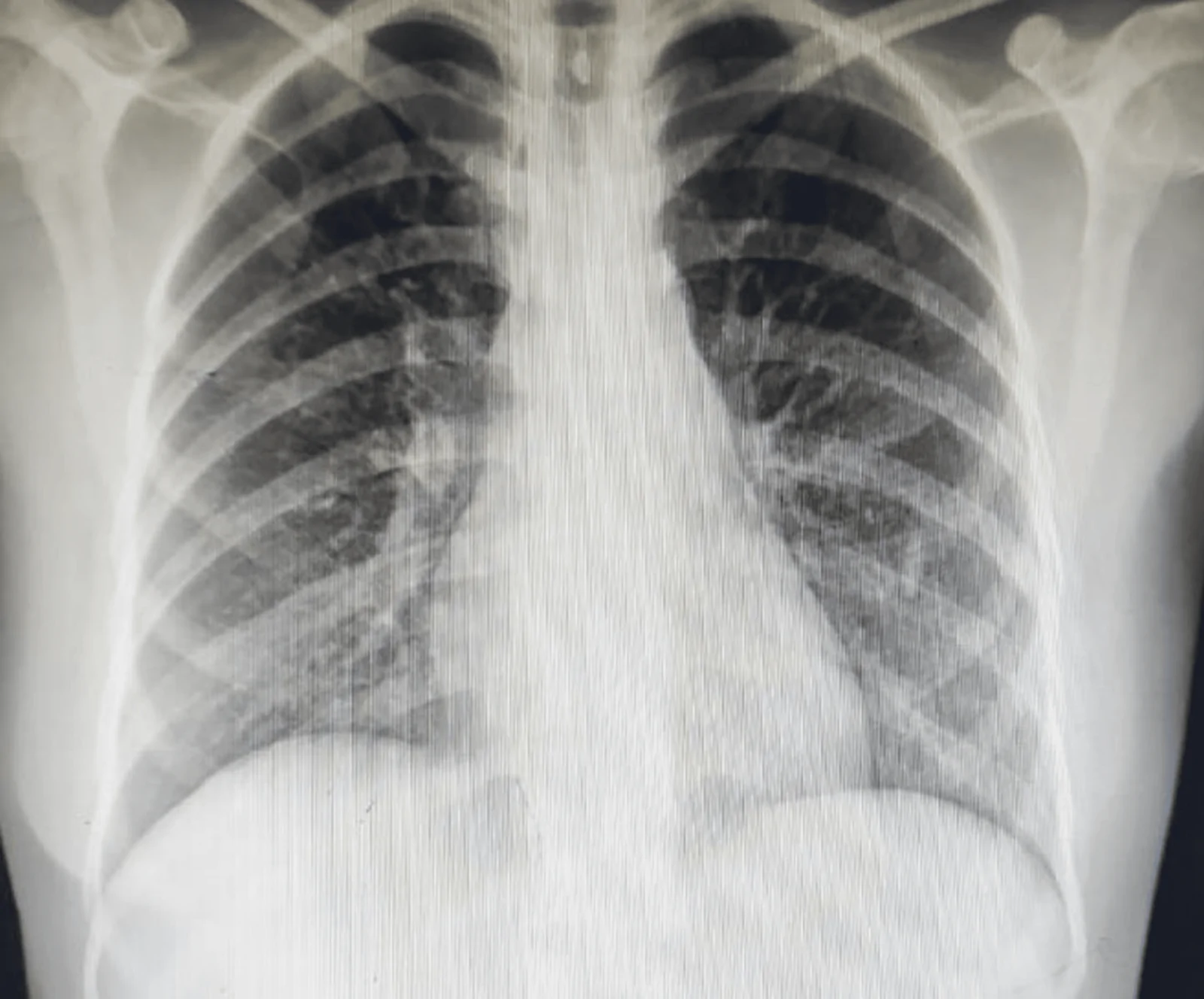
Chest X-ray of the patient

A 46-year-old female presented with fever, yellowish discoloration of the eyes, hematemesis, and melena for the past 7 days. Three months previously, she had a diagnosis of rheumatoid arthritis and was commenced on leflunomide 20 days before the onset of her current symptoms (designated as day 0). The lung X-ray shown in Figure 1 reveals significant findings that correlate with the patient's respiratory symptoms.

The clinical timeline progressed linearly from the drug's initiation. Fifteen days prior to presentation (Day +5 post-leflunomide), she developed an insidious-onset, high-grade fever accompanied by generalized tiredness. This was subsequently associated with mild, diffuse abdominal pain unrelated to feeding, progressive yellowish discoloration of the eyes, and severe pruritus all over the body. Over the preceding two days (Days +18 to +19 post-leflunomide), her condition deteriorated, manifesting as mild confusion, altered mood, reversed sleep-wake cycle, and active melena.

On examination, the patient was conscious and oriented to time, place, and person. She reported mild confusion, an altered mood, and a reversed sleep-wake cycle over the preceding days, consistent with Grade I hepatic encephalopathy by West Haven criteria. However, in the absence of coagulopathy (INR 1.15), this did not meet criteria for acute liver failure. She had pallor and icterus. Dermatological evaluation revealed a generalized, highly pruritic, symmetric, exfoliating erythematous rash covering approximately 65% of her total body surface area (BSA). The rash involved the face, upper trunk, and extremities, accompanied by prominent facial and periorbital edema; oral and genital mucous membranes were completely spared. On palpation, prominent, mobile, non-tender lymph nodes measuring 2 cm were noted in the bilateral axillary and cervical regions. Abdominal examination revealed significant epigastric tenderness; however, there was no clinical evidence of hepatomegaly or splenomegaly on palpation. Initial investigations are shown and summarized in Table 1.

**Table 1:** Initial laboratory investigations

Investigation	Value
Hemoglobin	7.1 g/dL
Hematocrit	21.8%
Total leucocyte count	4.7 x 10^3/uL
Platelet count	37 x 10^3/uL
Eosinophils	24%
Total bilirubin	9.16 mg/dL
Direct bilirubin	8.29 mg/dL
Aspartate aminotransferase (AST)	521 IU/L
Alanine aminotransferase (ALT)	>700 IU/L
Alkaline phosphatase	107 IU/L
International normalized ratio (INR)	1.15

Initial laboratory investigations upon presentation demonstrated severe pancytopenia, marked transaminitis, and hyperbilirubinemia (Table 1). In the absence of a prolonged coagulopathy (INR 1.15), the patient did not meet the strict criteria for acute liver failure. To rule out competing etiologies, a comprehensive infectious and autoimmune panel was performed. Hepatitis A, B, C, and E serologies and an antinuclear antibody (ANA) profile were negative. Polymerase chain reaction (PCR) assays for human herpesvirus 6 (HHV-6), Epstein-Barr virus (EBV), and cytomegalovirus (CMV) DNA were also negative, and multiple blood cultures remained sterile after 72 hours. A baseline chest radiograph (Figure 1) was performed due to mild respiratory discomfort, revealing significant findings of bilateral interstitial infiltrates and mild patchy opacities that correlated with pulmonary involvement.

A diagnosis of Drug Reaction with Eosinophilia and Systemic Symptoms (DRESS) was established and validated using internationally standardized diagnostic criteria. Applying the strict RegiSCAR validation scoring system, the patient achieved a final score of 8 out of 9, cementing a 'Definite' diagnosis of DRESS (Table 2) [7]. Leflunomide was stopped immediately. Because of the drug's exceptionally long half-life, an emergency accelerated elimination protocol using oral cholestyramine washout (8 g three times daily) was immediately initiated alongside an intravenous N-acetylcysteine infusion and supportive care.

**Table 2:** RegiSCAR validation score evaluation Note: A RegiSCAR score greater than 5 establishes a definite diagnosis [7].

RegiSCAR Criteria Component	Patient Findings & Clinical Presentation	Points Assigned
Fever (≥ 38.5°C)	Documented high-grade fever 15 days prior to presentation.	0 (Avoids -1 penalty)
Enlarged Lymph Nodes	2 cm nodes in 2 distinct sites (Axillary and Cervical).	+1
Atypical Lymphocytes	Present on peripheral blood smear with plasmacytoid features.	+1
Eosinophilia	Severe peripheral eosinophilia quantified at 24%.	+2
Skin Rash Extent	Widespread maculopapular eruption covering 65% BSA (>50% threshold).	+1
Skin Rash Morphology	Morbilliform progression accompanied by signature facial edema.	+1
Internal Organ Involvement	1 organ system involved (Grade 1 Hepatic Encephalopathy).	+1
Alternative Causes Excluded	Negative viral panels (Hep A/B/C/E, HHV-6, EBV, CMV), ANA, and cultures.	+1
Resolution Timeline	Clinical course timeline pending/not utilized for calculation.	0
TOTAL RegiSCAR SCORE	8 / 9	Definite DRESS Case

Over the next few days, her ferritin peaked at 9390 ng/mL, and she developed pancytopenia with hemoglobin 6.1 g/dL, platelets 11 x 10^3/uL, and a total leukocyte count of 1.3 x 10^3/uL.

Bone marrow aspiration and biopsy showed features of hemophagocytosis, confirming a diagnosis of HLH secondary to DRESS syndrome. A diagnosis of leflunomide-induced severe cutaneous adverse reaction, acute liver failure, and HLH was made. 

In addition to the RegiSCAR framework, the patient’s clinical course was cross-referenced against the strict Japanese Consensus Group (J-SCAR) diagnostic criteria for Drug-Induced Hypersensitivity Syndrome (DIHS/DRESS) (Table 3) [8]. The presentation satisfied 6 out of 7 core clinical parameters, meeting the classification for atypical DIHS/DRESS. The disease onset occurred exactly 20 days following the initiation of leflunomide, falling precisely within the mandated 2-to-8-week diagnostic incubation window. The patient demonstrated the hallmark clinical triad of the syndrome, characterized by a severe exfoliating cutaneous eruption, prominent hematologic abnormalities (24% peripheral eosinophilia alongside atypical lymphocytosis), and visceral organ injury manifesting as severe transaminitis (ALT >700 IU/L) and Grade I hepatic encephalopathy. Furthermore, the clinical and hematologic parameters continued to deteriorate systematically despite the immediate cessation of the culprit drug, fulfilling the J-SCAR requirement for a prolonged clinical course. Polymerase chain reaction (PCR) assays for human herpesvirus 6 (HHV-6) DNA were initially negative, a finding highly consistent with early-stage sample collection before late-phase viral reactivation typically occurs.

**Table 3:** Japanese Consensus Group (J-SCAR) criteria evaluation Note: The J-SCAR system criteria require 7/7 parameters for "Classic DIHS" and 5/7 parameters for "Atypical DIHS/DRESS" [8]. This case confidently clears the threshold for diagnostic validation. DIHS: Drug-Induced Hypersensitivity Syndrome; DRESS: Drug Reaction with Eosinophilia and Systemic Symptoms

J-SCAR Diagnostic Criterion	Patient Findings & Clinical Status	Status
1. Maculopapular rash developing >3 weeks after starting drug	Developed a generalized, exfoliating erythematous rash exactly 20 days post-leflunomide initiation.	Fulfilled
2. Prolonged clinical symptoms after discontinuing the culprit drug	Liver functions and blood counts continued to deteriorate systematically after leflunomide was withdrawn.	Fulfilled
3. Fever (> 38°C)	Documented high-grade, insidious-onset fever 15 days prior to presentation.	Fulfilled
4. Liver abnormalities (ALT > 100 U/L)	Severe transaminitis present with alanine aminotransferase (ALT) >700 IU/L, aspartate aminotransferase (AST) 521 IU/L, and Grade I Hepatic Encephalopathy.	Fulfilled
5. Leukocyte abnormalities (Leukocytosis, Atypical lymphocytes, or Eosinophilia)	Co-existence of marked peripheral eosinophilia (24%) and prominent atypical lymphocytes on blood smear.	Fulfilled
6. Lymphadenopathy	Confirmed 2 cm enlarged bilateral axillary and cervical lymph nodes.	Fulfilled
7. HHV-6 Reactivation	Early-stage viral PCR testing returned negative results.	Not Fulfilled
DIAGNOSTIC CONCLUSION	Satisfies 6 out of 7 core criteria parameters.	Atypical DIHS/DRESS

To formally validate the secondary HLH diagnosis, the patient's clinical markers and metabolic profile were cross-referenced against the HLH-2004 criteria framework (Table 4) [9]. The patient strictly fulfilled 5 out of the 8 core HLH-2004 criteria parameters, demonstrating a persistent high-grade fever, profound pancytopenia, marked hyperferritinemia peaking at 9,390 ng/mL, biopsy-confirmed bone marrow hemophagocytosis, and hypertriglyceridemia (242 mg/dL) paired with hypofibrinogenemia (160 mg/dL).

**Table 4:** HLH-2004 diagnostic criteria evaluation HLH-2004 diagnostic criteria [9]

HLH-2004 Criterion	Patient Findings & Laboratory Status	Status
1. Fever	Documented high-grade, insidious-onset fever.	Fulfilled
2. Splenomegaly	Noted as absent on clinical evaluation.	Not Fulfilled
3. Cytopenias (≥ 2 lineages)	Pancytopenia: Hb 6.1 g/dL, WBC 1.3 × 10³/µL, Platelets 11 × 10³/µL.	Fulfilled
4. Hypertriglyceridemia and/or Hypofibrinogenemia	Confirmed: Triglycerides 242 mg/dL (≥ 177 mg/dL) and Serum Fibrinogen 160 mg/dL (≤ 150 mg/dL or 1.5 g/L).	Fulfilled
5. Hemophagocytosis	Confirmed via histopathological bone marrow aspiration and biopsy.	Fulfilled
6. Low/Absent NK Cell Activity	Not performed / Unavailable.	Unknown
7. Hyperferritinemia	Marked serum ferritin elevation peaking at 9,390 ng/mL (≥ 500 ng/mL).	Fulfilled
8. Elevated Soluble CD25	Not performed / Unavailable.	Unknown
DIAGNOSTIC CONCLUSION	Fulfills 5 out of 8 core criteria parameters.	Definite HLH Diagnosis

Because the pediatric-centric HLH-2004 criteria can occasionally lack sensitivity in adult cohorts, the adult-specific HScore was calculated to offer further diagnostic objectivity (Table 5) [10]. The patient scored a total of 231 points, significantly exceeding the validated multi-center diagnostic cutoff of 169 and corresponding to an estimated diagnostic probability of reactive hemophagocytic syndrome greater than 98%. This dual-metric calculation definitively substantiates the secondary HLH diagnosis, documenting the intense systemic hyperinflammation and macrophage activation secondary to leflunomide-induced DRESS.

**Table 5:** HScore point breakdown for adult reactive hemophagocytic syndrome HScore criteria [10]

HScore Parameter	Patient's Clinical / Laboratory Value	Assigned Points
Known Immunosuppression	No prior history of chronic immunosuppressive therapy.	0
Maximum Temperature	High-grade fever (38.4°C - 39.4°C).	33
Organomegaly	No hepatomegaly or splenomegaly.	0
Number of Cytopenias	Pancytopenia (Anemia, leukopenia, and thrombocytopenia).	34
Serum Ferritin Level	9,390 ng/mL (Exceeds maximum scoring threshold of >6,000).	50
Aspartate Aminotransferase (AST) Level	521 IU/L (Exceeds maximum scoring threshold of ≥ 30).	19
Serum Triglycerides	242 mg/dL (Equivalent to 2.73 mmol/L; clears threshold of >1.5).	30
Serum Fibrinogen	160 mg/dL (Equivalent to 1.6 g/L; clears threshold of ≤ 2.5).	30
Bone Marrow Hemophagocytosis	Confirmed features on aspiration and biopsy.	35
TOTAL HSCORE	231 (Diagnostic Cutoff ≥ 169)	Probability of HLH > 98%

The patient was started on dexamethasone 16 mg IV daily and etoposide 200 mg IV as per the HLH-94 protocol. The patient required multiple blood product transfusions, as shown in Table 6.

**Table 6:** Blood product transfusions

Date	Product
Day 1	1 unit Packed Red Blood Cells (PRBC)
Day 3	2 units Random Donor Platelets (RDP)
Day 5	1 unit Cryoprecipitate
Day 8	2 units PRBC, 6 units RDP
Day 10	2 units PRBC

Over the next 2 weeks, her clinical status gradually improved with a reduction in bilirubin levels and rising blood counts, though she continued to have leukocytosis. Her rash and pruritus also resolved with topical steroids, details of which are provided in Table 7:

**Table 7:** Trend of complete blood counts

Date	Hemoglobin (g/dL)	TLC (x10^3/uL)	Platelets (x10^3/uL)
Day 1	7.1	4.7	37
Day 2	6.4	1.3	18
Day 3	7.6	1.3	17
Day 4	7.3	0.5	13
Day 5	7.4	0.7	16
Day 6	8.2	2.1	16
Day 7	7.5	4.1	21
Day 8	6.2	4.5	11
Day 9	7.7	5.8	27
Day 10	7.2	11.5	100
Day 11	7.4	22.5	275
Day 12	6.9	23.4	388
Day 13	8.7	24.5	493
Day 14	8.7	27.9	588

At discharge after 3 weeks, her aspartate aminotransferase (AST) was 49 IU/L, alanine aminotransferase (ALT) 164 IU/L, total bilirubin 11.8 mg/dL, and hemoglobin 7.5 g/dL. She was discharged on a tapering course of oral steroids and advised close follow-up.

## Discussion

Leflunomide is associated with a 3-6% risk of elevated liver enzymes, though clinically apparent drug-induced liver failure is very rare (<1%) [11]. DRESS syndrome due to leflunomide occurs in <0.5% of cases. Leflunomide, an immunomodulatory agent used for treating rheumatoid arthritis and other autoimmune conditions, is generally well-tolerated but carries the risk of severe adverse reactions. The incidence of elevated liver enzymes with leflunomide use is reported to be between 3-6%, but clinically significant acute liver failure remains exceedingly rare, occurring in less than 1% of cases [12]. Furthermore, leflunomide has also been linked to DRESS syndrome. That is also an infrequent event, as less than 5% of patients taking the drug experience it [13]. The simultaneous occurrence of these side effects, concomitant with HLH, thereby requires intensive immunosuppression and, hence, has a lethal outcome.

A rare, life-threatening condition, DRESS syndrome stands for Drug Reaction with Eosinophilia and Systemic Symptoms [14]. It is a severe hypersensitivity reaction characterized by a widespread rash, fever, enlarged eosinophils in the blood, and internal organ involvement, typically the liver. As drug-specific T-cells would be activated and undergo clonal expansion before their cytotoxic effects, the pathogenesis involves a complex interplay among genetic predisposition, drug metabolism, and immune response leading to cytokine storm. Such a hyperinflammatory state can cause additional complications, such as HLH, a disorder of uncontrolled immune activation, with overwhelming systemic inflammation, multi-organ failure, and very high mortality if left untreated.

Infections, malignancies, autoimmune diseases, and certain medications, like leflunomide, can incite HLH [15]. One of the defining features of HLH is the presence of fever and cytopenias along with hepatosplenomegaly, hyperferritinemia, and hemophagocytosis on bone marrow biopsy [16]. It is usually diagnosed by fulfilling diagnostic criteria based on clinical, laboratory, and histopathological findings.

An increased blood test result would be atypical for the potential for DRESS syndrome [17]. However, the presence of fever, jaundice, melena, and a widespread exfoliating rash accompanied by findings of direct hyperbilirubinemia, transaminitis, eosinophilia, and pancytopenia seems to suggest DRESS syndrome, and the diagnosis was eventually confirmed as leflunomide-induced severe drug-induced liver injury with Grade I hepatic encephalopathy. Bone marrow showed hemophagocytosis, and serum ferritin was also markedly elevated, confirming the subsequent development of HLH [18]. The key to treating HLH is early administration of immunosuppressive therapy to stop the hyperinflammation. Currently, the most recommended protocol for treatment is the HLH-94 protocol, which contains dexamethasone and etoposide [19]. The patient received this therapy and supportive measures (blood product transfusions), and clinical and laboratory parameters gradually improved.

This case is consistent with observations that leflunomide-induced liver injury in Indian patients tends to present with severe cutaneous involvement rather than the more indolent hepatocellular pattern typically described in Western cohorts and contributes additional detail on the natural history of this regional phenotype when complicated further by secondary HLH [20]. A diagnosis of DRESS was confirmed, with a RegiSCAR score of 8/9, rising to a maximum of 9/9 due to progressive hepatic and pulmonary involvement. The patient's condition, induced by leflunomide, fulfilled J-SCAR criteria and was further complicated by secondary HLH, which was confirmed via bone marrow biopsy showing hemophagocytosis. Treatment included dexamethasone and etoposide following the HLH-94 protocol.

The present case highlights the early identification and appropriate management of drug-induced severe adverse reactions. For patients who present with systemic symptoms and cytopenias, clinicians should continue to have a high index of suspicion for DRESS syndrome and HLH, especially in the setting of a new medication such as leflunomide. Prompt withdrawal of the causative agent and initiation of treatment can be life-saving. The case also highlights the importance of monitoring these patients long-term and offering supportive care in cases of severe reactions. These patients need to be followed carefully post-discharge, even following clinical improvement, to manage secondary infections due to immunosuppression and possible relapses of HLH.

## Conclusions

This case report illustrates a rare but critical instance of leflunomide-induced Drug Reaction with Eosinophilia and Systemic Symptoms (DRESS) syndrome progressing to secondary hemophagocytic lymphohistiocytosis (HLH). This combination presents significant diagnostic and therapeutic challenges. The patient, a 46-year-old female with a recent diagnosis of rheumatoid arthritis, exhibited rapid onset of systemic symptoms following the initiation of leflunomide. This led to a complex clinical scenario featuring severe drug-induced liver injury with Grade I hepatic encephalopathy. To satisfy formal diagnostic validation, objective scoring systems were utilized. This confirmed a definitive diagnosis of DRESS with a maximum RegiSCAR score of 9, alongside a highly probable secondary HLH diagnosis supported by an adult HScore of 231 (estimated probability >98%) and 5 out of 8 fulfilled HLH-2004 criteria. ​​​​​​​Clinical stabilization and recovery were associated with a prompt, multimodal therapeutic approach. This strategy relied heavily on the immediate cessation of leflunomide and the administration of N-acetylcysteine infusion, systemic corticosteroids, blood product transfusions, and targeted components of the HLH-94 protocol.

This case underscores the absolute necessity for healthcare providers to maintain a high level of vigilance for severe systemic drug reactions and secondary hyperinflammatory syndromes when patients present with cutaneous eruptions, transaminitis, and cytopenias soon after starting high-risk medications like leflunomide. Prompt recognition, immediate drug withdrawal, and coordinated multidisciplinary treatment are vital to prevent catastrophic outcomes, mitigate potential relapses, and ensure patient recovery.
